# FlyClimber: a new user-friendly, automated method to measure *Drosophila* motor coordination

**DOI:** 10.1242/bio.062500

**Published:** 2026-05-28

**Authors:** Callan Sharples, Petros Ligoxygakis

**Affiliations:** Department of Biochemistry, University of Oxford, South Parks Rd, OX1 3QU, Oxford, UK

**Keywords:** *Drosophila*, Climbing assay, Automated methods

## Abstract

The climbing assay, used to assess locomotive capabilities of *Drosophila*, takes advantage of the negative geotropism reflex. After a vial is knocked at the bench and flies are brought down to the bottom by that movement, they exhibit the instinct of climbing up away from gravity. Measuring how high flies can climb (performance index) provides a measure of their locomotion. However, the performance index is only a rough estimation of average height, while manual data analysis is slow and prone to human error/bias. The method has been modernised, and semi-automated but current protocols have several drawbacks. Programs are data intensive, requiring the storage of many videos/photos with large file sizes. In this context, we produced FlyClimber, a user and set-up friendly Python-based program. FlyClimber requires minimal hardware set up, reducing the required data storage space while providing accurate and robust data with high sensitivity. Employment of Statistical Parametric Mapping is novel for climbing assays, providing a time series of data with more potential for analyses than can currently be achieved by existing programs or manual methods.

## Teaser

**Summary:** A new automated climbing assay with minimal set up to be used in *Drosophila* research.

## INTRODUCTION

Assays to study behaviour have been at the origins of *Drosophila* research since the experiments of F. W. Carpenter who studied fly mechano-sensation, phototaxis and geotaxis (Carpenter, 1905, for a review see Greenspan, 2008). Efforts continued in T. H. Morgan's group by R. S. MacEwan, focusing again on photo and geo tropism (MacEwan, 1918, 1925). Pioneering studies by Hirsch and coworkers linked individual differences in geotaxis to specific chromosomes opening the way for gene-level analysis (Erlenmeyer-Kimling and Hirsch, 1961). Work by Seymour Benzer established the genetic basis of many mechanisms underlying *Drosophila* behaviour and introduced the ‘counter current’ procedure as a test for adult phototaxis, suggesting it could be used with a wide variety of stimuli including gravity (Benzer, 1967). Barry Ganetzky built on the initial suggestion to study age-related traits including locomotion (Ganetzky and Flanagan, 1978). Measuring locomotion has also been used in the context of neurodegenerative disease modelling as it is considered akin to testing motor coordination (Feany and Bender, 2000).

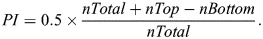


The assay takes advantage of the instinctive upwards movement of the flies (negative geotaxis) when they are gently tapped to the bottom of the cylinder or vial, they are contained. A camera records the flies as they climb upward. The number of flies that reach a certain distance from the bottom within a specified time limit is recorded and scored. The vial/cylinder is sectioned into unequal thirds with lines drawn at 3 cm and 10 cm from the bottom. The assay is performed over a 30 s period with manual timing and video recording. Flies are counted at 30 s in each section and passed through a formula calculating a performance index (PI).

This method has several drawbacks:

- It is a data intensive process requiring the storage of many large video/photo files.
- Data analysis is slow and prone to human error/bias even if experiments are blinded.
- As a rough estimation of average height, PI is inaccurate.
- Often only data at a single time point is collected.

Programs have been written to semi-automate the climbing assay and improve upon these drawbacks, with the most popular being the program FreeClimber (Spierer et al., 2021). FreeClimber drastically improves the climbing assay by calculating the exact height of flies in the vial at many time points, creating a time series dataset of fly height. However, unique hardware (often built in-house) is required for the program to function adequately, making it inaccessible to most labs including ours.

Considering this, we wanted to produce our own Python program that could be used with minimal set up, drastically reduce the storage space required for data, and provide accurate data in greater quantities, capable by manual methods. We call this program FlyClimber.

## RESULTS

### FlyClimber setup

**Fig. 1.**
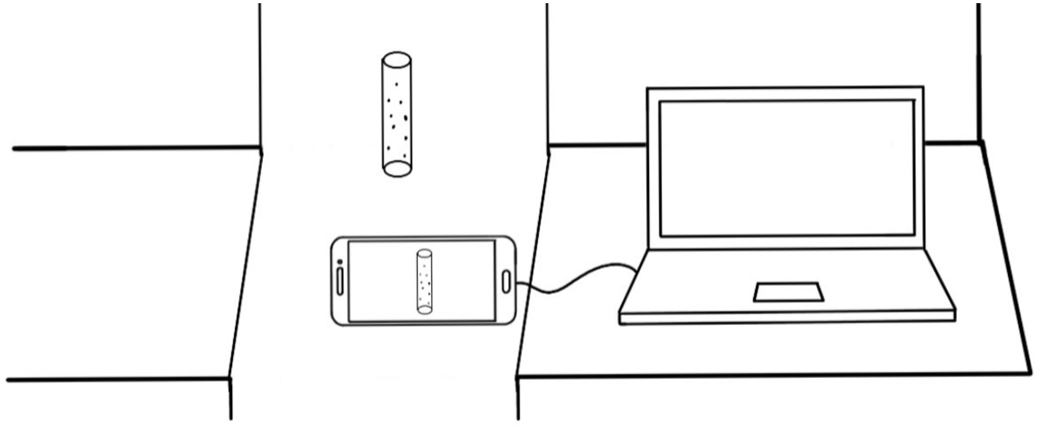
**Schematic of example setup.** Flies are placed in a vial in front of a white background. A phone camera is used for video capture, while a connected laptop runs the program for data collection.

The setup requirements for FlyClimber are minimal. The only requirement is that the assay is performed in front of a white background with a camera linked to the computer running the program. In our setup, this was achieved using a smartphone camera connected to a laptop via USB (Fig. 1).

### FlyClimber workflow

**Fig. 2.**
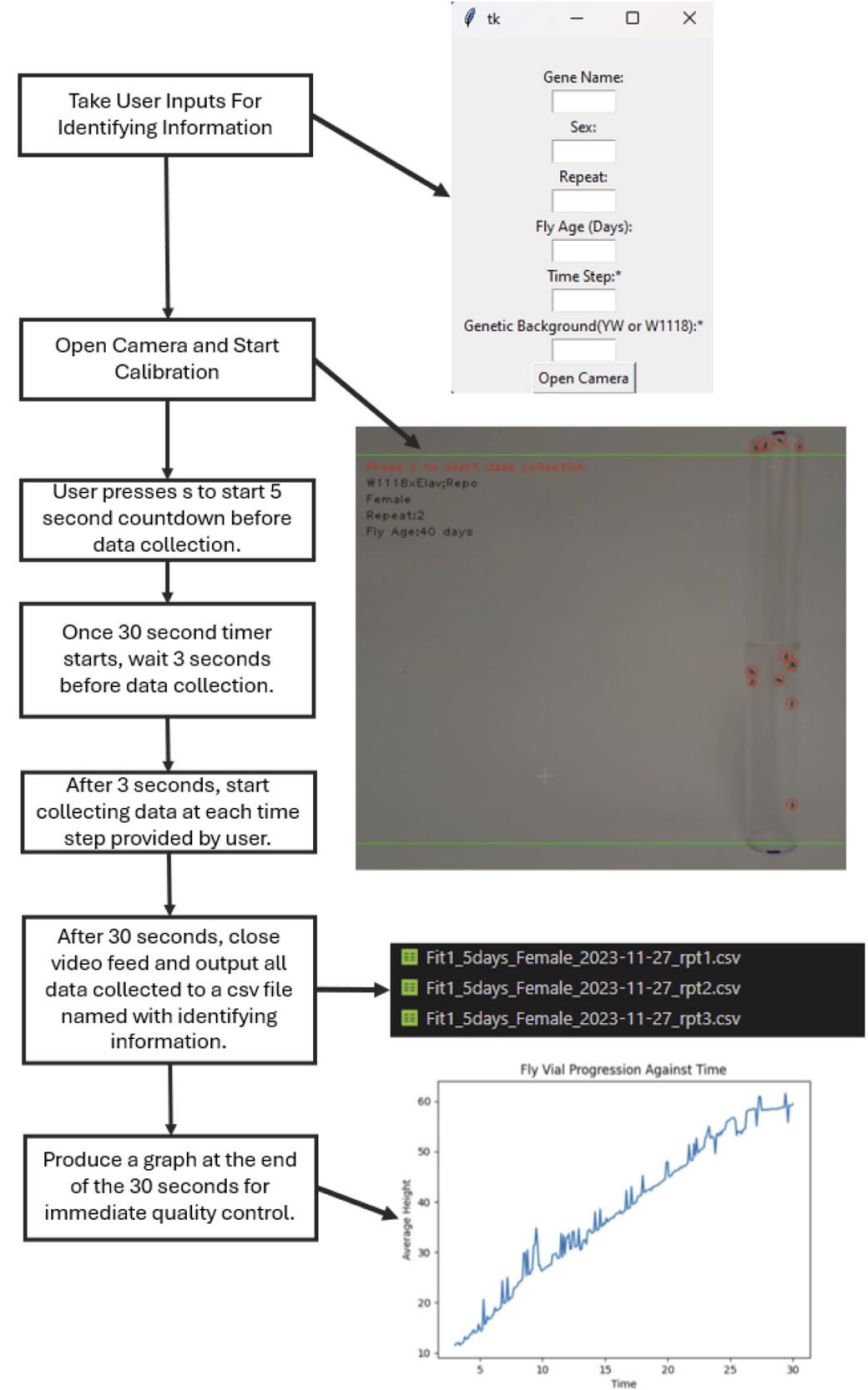
**Workflow outlining general methodology of FlyClimber.** The user inputs provide identifying information as to the genotype, sex, and age of flies, which experimental repeat is being carried out, the time step for frequency of data collection, and the genetic background so the program knows what type of fly is being tracked. The calibration phase allows the user to ensure the setup is appropriate and the program is functioning as expected. The program monitors the size of flies during the calibration phase for multiple fly detection during the experiment. For the program to function, the bottom blue line on the vial must be kept below the lower green line on the display and the top blue line kept above the upper green line. The data stored in the csv files is a list of all flies' percentage height in the vial at each time point so if the program picks up excessive noise and erroneously adds this to the data file, the values can be manually removed after the data is stored. Identifying information must be unique so that an existing file is not mistakenly replaced. A graph is produced at the end of the 30 s of observation for qualitative checking that the program functioned correctly. Images within the figure are outputs of FlyClimber at various stages of the assay.

The overall workflow for FlyClimber is shown in Fig. 2. It demonstrates a program that can collect data in real time, store it in small file sizes and provide immediate visualisation of the data for quality control.

### Image processing

The code of FlyClimber can be found in https://github.com/Ligoxygakis-Lab/FlyClimber. FreeClimber utilises background subtraction where pixels that do not change for the entire video are removed leaving behind only the pixels that change, which are identified as the flies. In contrast, FlyClimber utilises colour filtration as the detection system. For each frame of the video, a colour filter is applied, removing pixels not within a given colour range. Two filters are applied, a blue colour filter that detects the blue line drawn at the top and bottom of the vial. This allows the program to detect and track the confines of the vial. The other filter applied is specific to the colour of the flies. Clusters of filtered pixels are identified as flies. This can be very sensitive to noise. To solve this, various noise filtration methods are implemented.

### Multiple fly detection

Issues arise from the image processing in the case where multiple flies are in proximity. The flies appear as a single cluster resulting in loss of data from the additional flies. To solve this, a detection system is put in place that tracks the relative size of flies compared to the size of the vial during the calibration phase. During data collection, if a cluster larger than the expected size is found, it will calculate the number of flies based on the identified radius compared to the expected radius and add the percentage height to the dataset multiple times for each fly. Visual feedback is given to the user by circling multiple fly clusters in blue as opposed to red. Movie 1 is an example of how the FlyClimber software works.

### FlyClimber statistics

**Fig. 3.**
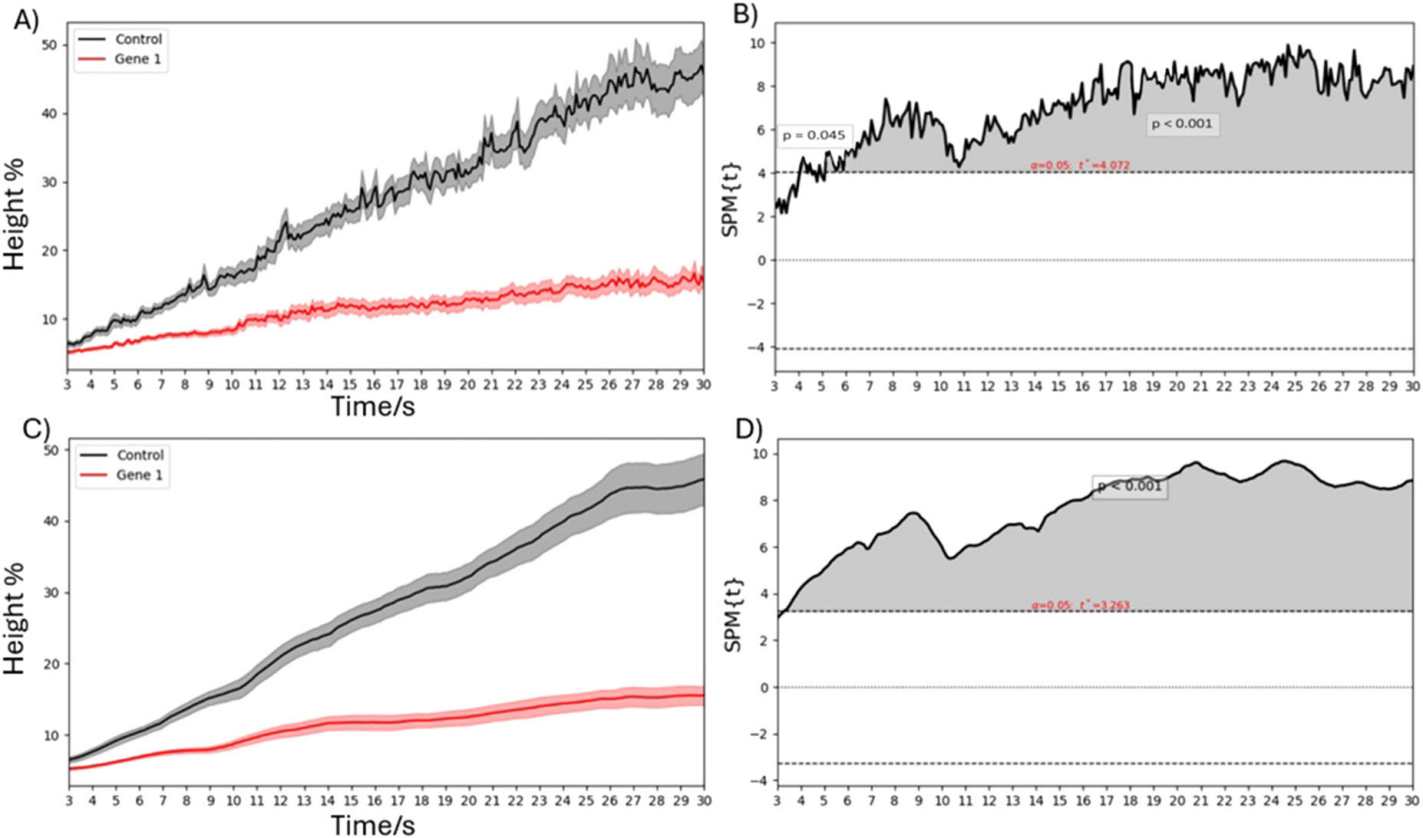
**A hypothetical example dataset showing the smoothing that can be applied.** (A) Shows raw unsmoothed data for control and gene1 with locomotion deficit. (B) SPM output for data in panel A. (C) Data post-smoothing with the overall trend in data preserved but noise removed. (D) SPM output for data in panel C. Shaded region in B and D indicate regions where statistical significance was found.

For all climbing assays, the time step would usually be set to 0.1 s, the minimum value that can be handled by the program, resulting in a total of 271 time/data points. At a statistical significance threshold of α=0.05, this leads to a substantial amount of type I errors with no corrections performing *t*-tests between two sets of data. With a simple Bonferroni correction, the inverse problem of a high rate of type II errors is seen due to the Bonferroni correction's conservative nature. The Bonferroni correction assumes multiple individual comparisons. This is not the case for FlyClimber data with comparisons between one-dimensional time series, where data at each time point is heavily influenced by the time points surrounding it. To solve this problem, Statistical Parametric Mapping (SPM) is employed (Flandin and Friston, 2019). SPM is designed to handle N-dimensional data as it relies on Random Field Theory (RFT) for multiple comparisons corrections, which are often based on a gaussian distribution. SPM uses RFT to assess the relatedness of data points to their neighbouring data points. Closer related data points require less stringent multiple comparisons correction while the inverse is true with less related data points. This makes SPM far less conservative than options like the Bonferroni correction. The output for SPM is a separate graph showing the results of *t*-tests at each time point with significance against time where clusters of time points that pass the significance threshold have a single calculated *P*-value (Fig. 3). This allows us to identify significance within specific time ranges allowing for more detailed analysis of behaviour. SPM is computed using the python package SPM1D (Pataky, 2012). Given search parameters for specific genotypes, FlyClimber finds all applicable csv files, calculates median values for each time point for each file, compiles all data into a single matrix and opens a user interface with the graph. The user can alter the level of smoothing applied to the data using a Savitsky-Golay filter before computing the SPM statistics. The data should be smoothed in such a way that the noise is reduced without impacting the overall pattern of fly movement (Fig. 3). For each time point, a *t*-test is carried out. To ensure data are normally distributed for the *t*-test, median fly height for each repeat is used as the metric for statistical testing.

### FlyClimber validation

**Fig. 4.**
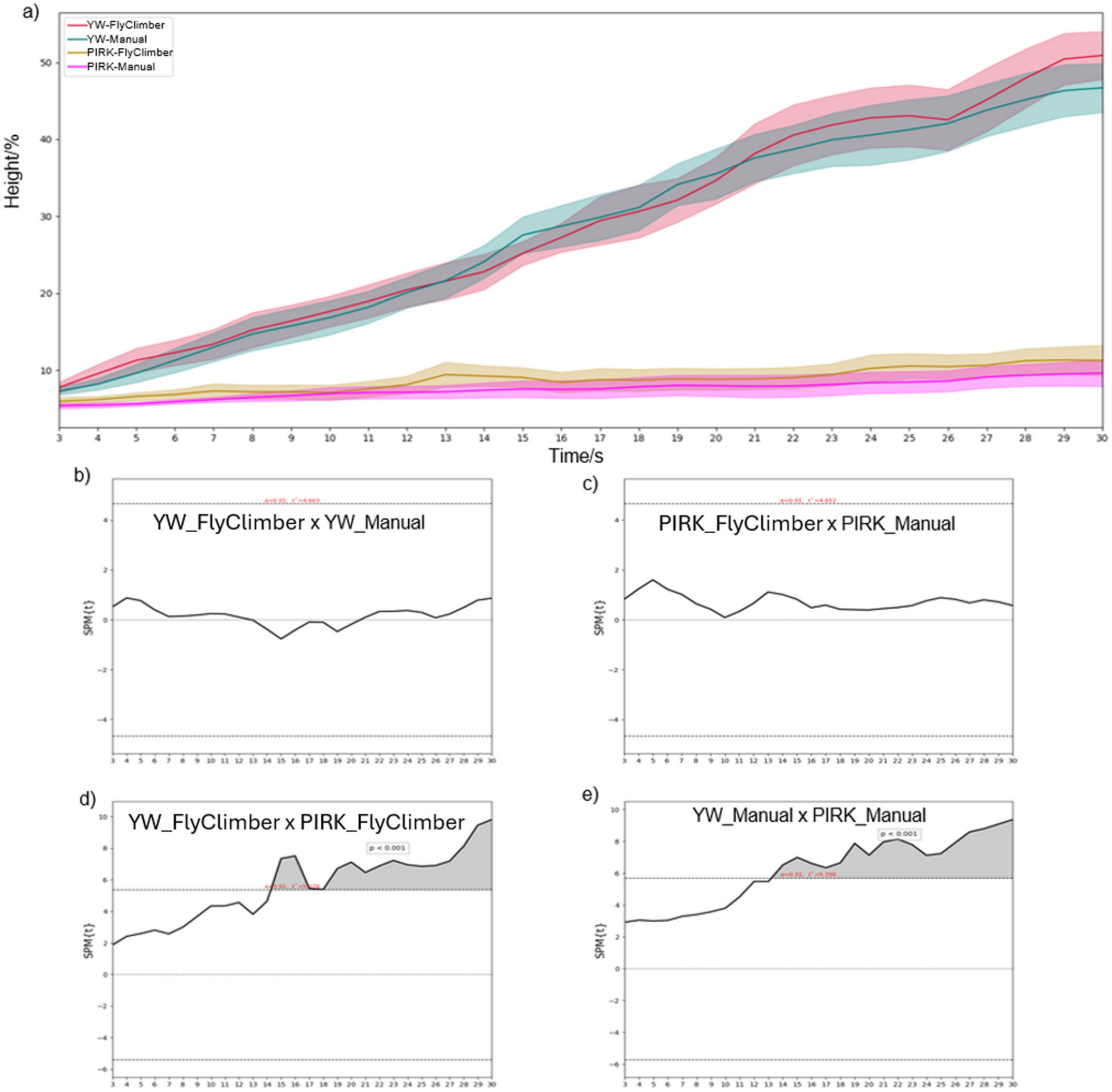
**Results from climbing assay performed on *yw* and *pirk*-knockout flies.** (A) Summary figure of data collected over seven technical repeats. Solid lines show average height progression of flies over time with the shaded region indicating standard error of mean. (B-E) Outputs for statistical parametric mapping performed. T value calculated by SPM (SPM {t}) at each time point is plotted. Shaded regions indicate that data has crossed the significance threshold indicated by the dotted line.

To validate FlyClimber we compared results obtained with this method with results through the classic ‘manual’ climbing assay (Ganetzky and Flanagan, 1978; Cao et al., 2013). To perform manual validation, the time step for data collection was set to 1 s and each time the program collected data it also saved a screenshot of the video frame when data was collected. For each screenshot the flies were manually identified and selected. Their true percentage heights were compared to the data collected by FlyClimber's detection (Fig. 4). This was done for 25-day-old control *pirk*-knockout flies previously shown to have a reduction in climbing performance with age (Kounatidis et al., 2017; Arora et al., 2025) and its *yw* genetic background.

There was no statistical difference for either *pirk* or *yw* flies between FlyClimber and manual detection indicating that FlyClimber is as accurate as the manual method in its tracking of flies. For both FlyClimber and manual methods SPM showed a statistically significant reduction in climbing ability of *pirk* compared to *yw* flies. This reduction was statistically significant from 14-30 s with *P*<0.001 in both methods (Fig. 4). This statistical difference was found even with the limited data set of seven technical repeats and a 1 s time step. FlyClimber is capable of up to a 0.1 s time step, drastically increasing the quantity of data and power of the statistical test. The time step was set to 1 s and only seven technical repeats were carried out due to the time intensive nature of the manual validation process.

FlyClimber has a tendency for low performing flies to over-report median fly height. Low performing flies are much closer together as they cannot spread out through the entire vial. This leads to FlyClimber missing some of the lower performing flies despite the multiple fly detection and a higher median value. As shown in Fig. 4, with a low time step and multiple repeats it is still a statistically insignificant difference.

## DISCUSSION

Previously published programs have sought to automate fly climbing (FreeClimber, FlyTracker) or unmarked animal movement in general (idtracker.ai, Romero-Ferrero et al., 2019). All are excellent in the automation of data analysis for climbing assays. They are capable of accurate, high throughput video processing to track flies' relative height in climbing assays at many time points. In FreeClimber, maximum speed of climbing is reported as their metric for locomotion through linear regression of the time series to identify the steepest portion of the graph, while idtracker.ai uses a convolutional neural network to follow the tracks of individual flies and thus derive both maximum speed as well as the trajectory. Finally, all three programs can track multiple flies simultaneously in the same vial, which is important as fly behaviour is different when they are individually tested versus tested as part of a group (Rooke et al., 2020).

While these programs have made advancements towards automation of the climbing assay, they do have limitations. They both detect flies by using background extraction of videos. For this to be viable it can only be used on prerecorded (not live) videos. Moreover, with idtracker.ai, the videos need to be pre-processed to label individuals prior to initial animal interaction analysis. However, climbing assays require individual trials to be carried out leading to many large video files to be stored. Quality of analysis is directly dependent on the quality of the videos taken, with specialist hardware yielding the best results. These setups are not common, and for researchers not in a neurobiology or behavioural research environment, this limits data analysis capability.

FlyClimber was created with the intention of being a versatile program that can be used with minimalist set ups and equipment while not requiring the need to store many large video files. By employing colour filtration of video frames along with various noise filtration mechanisms, these goals were achieved. Moreover, FlyClimber is capable of live video feed analysis, negating the need to record videos. The only files stored by the program are the output data csv files, which are very small in size. Because colour filtration of frames is used, markers placed at the top and bottom of the vial allow FlyClimber to not only track flies but also track the position of the vial in the frame. This allows FlyClimber to be robust at handling movement of the vial and flies within the frame due to camera movement. The same cannot be said for FreeClimber/idtracker.ai, where the vial boundaries must be manually defined for the program and are static throughout the data analysis for that video. Any movement of the camera or apparatus will result in a shift of the vial in the video and skew the data analysis.

Regarding data analysis, FreeClimber's metric of maximum climbing speed offers little insight into the original time series data and behaviour. By implementing SPM into FlyClimber, statistical insight into fly performance across all time points is possible. Providing contextual data allows for a much more detailed analysis and conclusions. Examples of contextual data include flies reaching the same height, which may have taken different lengths of time to reach that position, or two groups of flies that started climbing at the same speed but may stop climbing at different points. SPM offers the greatest potential for conclusions to be drawn and is streamlined by FlyClimber to sort and filter through csv files automatically given user input search parameters.

FlyClimber is a unique user and set-up friendly program that is robust while still maintaining high accuracy and sensitivity. Its employment of SPM is novel for climbing assays, providing more data and potential for analyses than can currently be achieved by existing programs or manual methods.

## MATERIALS AND METHODS

### Fly strains

The *pirk* mutant (*pirk^EY00723^*) (Lhocine et al., 2008) was obtained from Bruno Lemaitre and *yw* from the Bloomington *Drosophila* Stock Centre (BDSC #1495).

### Generation of flies used during validation

Three batches of six to eight females and three males were taken from the stocks for both *pirk* and *yw*. These flies were allowed to reproduce in separate vials until larvae were observed in the food, at which point the parent flies were removed. Once flies started emerging from pupae, all vials were cleared of any flies that had already hatched. 48 h later all flies that had hatched were collected and used for manual validation experiments. All *yw* flies were kept, while *pirk* flies needed to be selected against the Cyo balancer chromosome to ensure homozygous knockout. During the 25-day aging, male and female flies were kept together. 24 h before testing, 15 female flies for both genotypes were separated using CO_2_. Only female flies were tested on as previous tests using other methods had shown a greater reduction in locomotion for female flies compared to male flies for pirk.

### Climbing assay

Fly locomotion was assessed using the climbing assay. Upon knocking a vial of flies on the bench to knock them all to the bottom, they exhibit negative geotaxis, the instinct to start climbing upwards. Evidence for locomotive ability being dependent on the neurologic state of the fly (Barone and Bohmann, 2013), makes the climbing assay popular for assessing neurological decline. For each assay, 15 flies were transferred into an empty 1.5 cm×14 cm vial. The top and bottom of the vial were marked with a blue line. After transferring to the empty vial, the flies were allowed to acclimatise to their environment for 30 min before being placed in front of a white background. The vial was banged on the bench three times with roughly equal force each time and data were collected over the following 30 s. A 10 min rest was given to flies before the experiment was repeated to generate technical repeats. Data were collected using FlyClimber at https://github.com/Ligoxygakis-Lab/FlyClimber. Detailed instructions on using the program are contained within the README file in the Github repository.

### Data analysis

FlyClimber was used for data analysis. SPM with *t*-tests on the median value was used for statistical testing. Data were smoothed using a Savitzky-Golay Filter before SPM to remove noise. Smoothing was kept to a minimum to preserve the overall trend in the data in order to not affect the results of SPM. For multiple genotypes with a common control, a Sidak multiple comparisons correction was applied to the significance threshold.

## Supplementary Material

10.1242/biolopen.062500_sup1 Supplementary information
