# Supplementary figures and images for: FlyClimber: a new user-friendly, automated method to measure Drosophila motor coordination
Source: Biol Open. 2026 May 28;15(5):bio062500. doi: 10.1242/bio.062500 (PMC13267769; doi:10.1242/bio.062500)

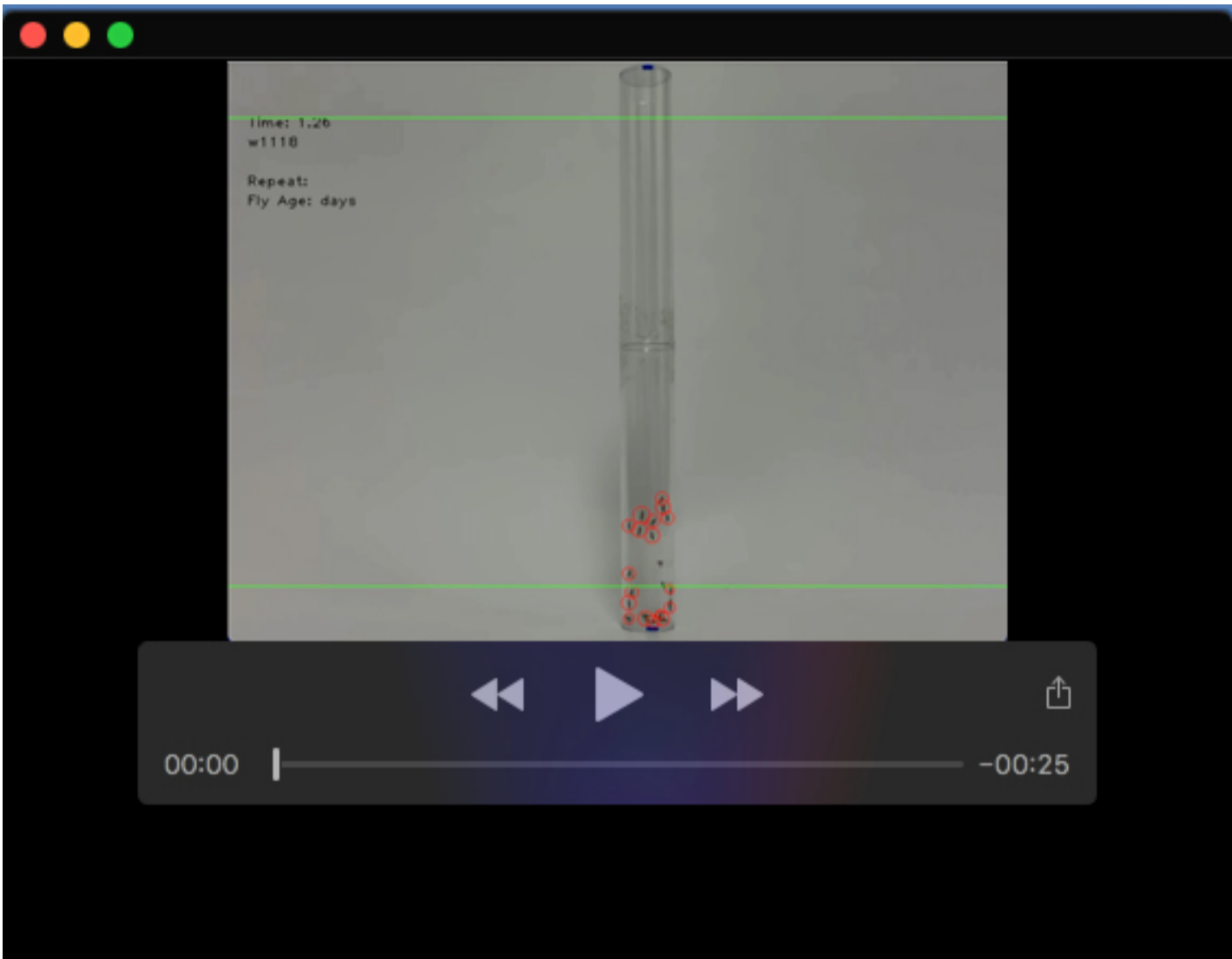

**Movie 1.**

Supplement: Supplementary information [file biolopen-15-062500-s1.pdf]
